# Developing a prognostic model for skin melanoma based on the persistent tumor mutation burden and determining IL17REL as a therapeutic target

**DOI:** 10.1007/s00432-024-05843-x

**Published:** 2024-06-20

**Authors:** Mingze Xu, Xinyi Ma, Yuchong Wang, Ziqin Yu, Xiaoli Zheng, Haiying Dai, Chunyu Xue

**Affiliations:** 1 Department of Plastic Surgery, Changhai Hospital, Naval Military Medical University, 168 Changhai Road, Shanghai, 200433 People’s Republic of China; 2 Department of Radiology, Changhai Hospital, Naval Military Medical University, Shanghai, China; 3 Basic Medical School, Southwest Medical University, Luzhou, Sichuan China

**Keywords:** Cutaneous melanoma, Prognostic model, Persistent tumor mutation burden, Therapeutic target, Gene signature

## Abstract

**Background:**

One popular and well-established marker for the immune checkpoint blockade (ICB) response is tumor mutation burden (TMB). Persistent TMB (pTMB), a subset of TMB, provides a better indicator to predict patient ICB therapy outcomes, as shown by some studies. Immune checkpoint drugs have significantly changed how melanoma is treated in recent years.

**Methods:**

In this study, we integrated the TCGA-SKCM database and data of pTMB of TCGA from the paper that first mentioned pTMB and analyzed mutational and Immune characteristics associated with pTMB level in SKCM. Next, the predictive DEGs were identified the subgroups of pTMB by Cox regression and LASSO analyses to construct a pTMB-related signature. Finally, the expression and Biological functions of signature genes was detected, and further validated in vitro assay.

**Results:**

In the current research, we explored the mutational and immunological features related to the level of TMB in cutaneous melanoma (CM). The high-pTMB subgroup exhibited an increasing incidence of gene changes and higher levels of immune cell infiltration. Subsequently, we established a pTMB-related signature based on the predictive DEGs and found the biological features and immune-associated variables between two distinct risk groups. Lastly, the results of the clinical sample validation demonstrated that the expression of IL17REL was down-regulated in the collected samples of individuals with CM. The in vitro assay results indicated that IL17REL effectively suppressed the proliferation, clonality, and migration of CM cells.

**Conclusion:**

In conclusion, we have developed a prediction model associated with TMB and subsequently validated the potential influence of IL17REL on Overall Survival (OS) in patients diagnosed with melanoma.

**Supplementary Information:**

The online version contains supplementary material available at 10.1007/s00432-024-05843-x.

## Background

Skin cancer developing from melanocyte stem cells and fully differentiated melanocytes is known as cutaneous melanoma (CM), a highly aggressive dermal carcinoma (Centeno et al. 2023). Melanomas, including around 1 in 5 skin cancers, are estimated to have affected approximately 325,000 individuals worldwide in 2020. Skin cancers, the most frequently diagnosed category globally, are anticipated to be responsible for over 1.5 million new cases in 2020 (Arnold et al. 2022). In recent years, immune checkpoint inhibitors have brought about a significant paradigm shift in treating melanoma, particularly in cases of advanced melanoma (Serratì et al. 2022). The accepted standard adjuvant therapy for managing and treating CM (stage III or IV) is using an inhibitor of the programmed cell death protein − 1 (PD-1) (Patrinely et al. 2021; Carlino et al. 2021). Although better results have been linked to immune checkpoint blockade (ICB), roughly half of patients do not see long-term benefits (Jie et al. 2022). Various biomarkers, such as tumor neoantigen burden (TNB) (Luo et al. 2022) and tumor mutation burden (TMB) (Mcgrail et al. 2021), have been reported for utilization in predicting ICB response; however, the findings of these indicators do not reliably predict the clinical outcome of patients. Building innovative and reliable prediction technologies and tools is necessary for precise individual assessment and pre-selection of suitable therapies for patients.

The primary biomarker for identifying cancer patients who can benefit therapeutically from ICB is the high TMB (Jung et al. 2023). Within the tumor mutation burden framework, all mutations are considered to be of similar significance, with variations observed solely in terms of mutation quantity. In the context of immunogenicity, specific mutations exert greater influence than others (Leung and Mcgranahan 2023). Hence, it is not always feasible for TMB to consistently demonstrate clinical efficacy in predicting the response to cancer immunotherapy. The concept of persistent TMB (pTMB), which denotes mutations that always elicit immune tumor control throughout the progression of tumors, was initially introduced by Niknafs et al. (2023). Significantly, the study's authors highlighted that pTMB has superior predictive capabilities for tumor ICB response compared to TMB. This finding offers novel perspectives for the precise prognosis of patients with CM.

This study examines the pTMB features of melanoma patients by analyzing the data from the Gene Expression Database (GEO), the Cancer Genome Atlas Program (TCGA), and all relevant scientific data from research by Niknafs et al. We developed a signature for predicting melanoma prognosis and response to chemotherapy and immunotherapy using Cox-Lasso regression based on the discovered differential genes with prognostic importance, allowing for individualized patient treatment regimens.

## Materials and methods

### Samples of melanoma patients

The collection of clinical samples from the patients conformed to the requirements stated in the Declaration of Helsinki. Before donating tumor tissue, all patients provided their informed consent by signing the necessary documentation. The surgeries were performed based on clinical indications; only residual tumor material was contributed to the research. The sample of patients consisted of three individuals. Pathological biopsies were conducted to diagnose two cases.

Furthermore, extensive resection of the primary tumor was performed. The other patients were diagnosed during the surgery via frozen section pathology. A pathologist sectioned the surgically excised tumor tissues in the operation room. The western blotting assay was performed, for which the tumor and the normal tissue portions were collected and cryopreserved from the patients.

### Data collection and processing

The gene expression profile of TCGA- SKCM (log^2^ (FPKM + 1) conversion) was downloaded from the R package "TCGA biolinks". The TCGA official post-correction survival information (OS) and clinical data (including gender, age, grade, stage, etc.) were collected from a published work by Liu et al. (2018). The data of pTMB of TCGA was obtained from (Niknafs et al. 2023), which included 107 patients of SKCM (skin cutaneous melanoma). Moreover, the GSE65904 expression data set for model validation was retrieved from the GEO database (https://www.ncbi). Exclusion criteria were used for patients lacking survival information or had insufficient clinical data.

### Mutational characteristics associated with pTMB level in SKCM

The pTMB was divided into two subgroups, high-pTMB(H-pTMB) and low-pTMB(L-pTMB), and was defined by an optimal cutoff value. Subsequently, the Kaplan–Meier (KM) survival analysis was used for the prognosis of the two subgroups. Using the R "maftools" package, the waterfall plot of somatic mutations in the pTMB subtypes was generated. Additionally, the R "ggplot2" package was employed to create a correlation dot plot, illustrating the associations between pTMB and several factors, including TMB, TNB, homologous recombination deficiency (HRD), and chromosomal instability (CIN). The TMB, HRD, and TNB data for cutaneous melanoma were obtained from a study by Thorsson et al. (2018), and the CIN statistics were retrieved from the research done by Drews et al. (2022). Further examination was conducted to assess the correlation between various subgroups of pTMB and the clinicopathological characteristics of cutaneous melanoma.

### Immune characteristics associated with pTMB level in SKCM

A comprehensive investigation was conducted to assess the relationship between various pTMB groups and immunological status in SKCM patients. The calculations on the ESTIMATEScore, ImmuneScore, TumorPurity, and StromalScore of both the low- and high-pTMB subgroups were done using the R package "ESTIMATE" (estimation of stromal and immune cells in malignant tumor tissues using expression data). For the quantification of the relative frequencies of the types of cells that had infiltrated the tumor-immune microenvironment (TIME), a ssGSEA (single-sample gene set enrichment analysis) method was used. The gene set of the infiltrating immune cell types identified in each TIME was acquired from a study by Charoentong et al. (2017). The R package "GSVA" (gene set variation analysis) was used to compare immune pathways at various pTMB levels. To generate heat maps, the R package "ComplexHeatmap" was used.

### Establishing and validating the pTMB-related signature

The DEGs (differentially expressed genes) were identified between the subgroups of pTMB. The limma algorithm was used (*p* < 0.05). Subsequently, a univariate Cox regression analysis was conducted to determine the predictive DEGs. Using the "glmnet" package in R software, regression analysis was done by LASSO (Least Absolute Shrinkage and Selection Operator). This identified the signature genes and eliminated the overfitting issue. Patients' risk scores were determined by evaluating the level of expression for each prognostic gene with its related coefficient of regression.

$${\text{riskScore}} = \sum\limits_{i = 1}^{n} {\exp_{{\text{i}}} } * {\upbeta }_{{\text{i}}}$$

where exp_i_ represents the gene expression level, β_i_ indicates the estimated regression coefficient value, and n represents the number of signature genes.

Patients diagnosed with SKCM were classified into two categories, namely high-risk and low-risk groups. This was based on the respective median risk scores. Subsequently, the survminer (survival analysis and visualization) R package was used to evaluate the OS of high- and low-risk categories of SKCM-classified patients. The curve of time-dependent ROC (receiver operating characteristic) was determined using the R "survminer" and "timeROC" utilities. In addition, univariate and multivariate Cox analyses were conducted to determine the predictive risk scores and independent prognostic values. Moreover, the formula that calculated the risk score for cohort validation was also used here. Afterward, the multivariate and univariate Cox analysis assessed the risk score independence as a prognostic determinant for patients afflicted with cutaneous malignant melanoma.

### Differences in biological characteristics between the prognostic signature low- and high-risk groups

Based on a *p-*value cutoff of < 0.05, the prognostic markers of high-risk and low-risk groups' distinct pathways were evaluated, which was analyzed using the R "GSVA" package. Moreover, the heat maps were generated using the R "ComplexHeatmap" package.

### Establishment of a nomogram model and clinical correlation analysis

Based on age, sex, clinical stage, Breslow depth, and pTMB-related features, a nomogram was constructed. To increase the clinical validation value even further, the actual and expected probabilities of 1, 3, and 5-year OS have been determined using calibration curves. The discriminatory capacity of each component to SKCM was analyzed using the ROC.

To analyze the differential expression among the tumor and normal samples in SKCM, the association between RiskScore and clinical characteristics was examined. Additionally, signature genes' predictive value and clinical importance were assessed to validate and identify potential candidate genes.

### Immune-associated characteristic differences and assessment of the drug sensitivity

The study investigated the microenvironmental variations of tumors concerning the prognostic hallmark low- and high-risk subgroups using a ssGSEA method. Gene sets specifying TIME invading immune system cells were obtained explicitly from the research conducted by Charoentong et al. (2017). The 29 gene sets representing immunological properties were taken from a study by He et al. (2018). The enrichment level of immunological characteristics between low- and high-risk subgroups of predictive signature was then qualified using the ssGSEA algorithm. Following this, a systematic search was conducted for expression profiles of ICB genes that are accessible to the public and provide comprehensive clinical data. Thus, the study included two immunotherapy cohorts: one with metastatic melanoma patients receiving anti-PD- 1 antibody treatment (referred to as Cohort PRJEB23709 (Gide et al. 2019)) and another involving melanoma patients receiving anti-CTLA- 4 antibody intervention (referred to as Cohort phs000452.v2.p1).

SubMap compared expression profiles to determine treatment impact. Therefore, the SubMap algorithm predicted the probability of anti-PD- 1 and anti-CTLA- 4 therapy responses. The data and its associated annotations were from 47 cutaneous malignant melanoma patients from published research by Lux et al. (2017).

### Western blot

Tumor cells or tissues were taken from patients and subjected to protein extract. Normal cells and tissues (non-cancerous) were also collected for comparison. Proteins were extracted by radio immunoprecipitation assay (RIPA; Shanghai Life Mode Engineering, Shanghai, China) and phenylmethylsulfonyl fluoride (PMSF; Shanghai Life Mode Engineering, Shanghai, China). The BCA protein assay kit (bicinchoninic acid; Shanghai Dongsheng Biotechnology, Shanghai, China) assessed the extracted protein concentration.

The PVDF (polyvinylidene difluoride) membrane, of 0.22 μm size (Millipore ISEQ00010, USA), was incubated overnight at 4 °C with an anti-IL17REL (1:1000, Thermo Scientific). Subsequently, the membrane was incubated with a 1:2000 dilution of the secondary antibody conjugated with horseradish peroxidase (HRP; Abcam, Cambridge, UK). The detection and visualization of the protein were carried out using the Prime Western Blotting Detection Reagent (Cytiva, UK). The ChemiDoc MP imaging system (Tanon 4800, Shanghai, China) detected chemiluminescence, and the ImageJ software was used to analyze the bands' gray values.

### Real-time quantitative PCR (RT-qPCR)

Utilizing the TRIzol reagent (Invitrogen, Waltham, MA, USA), total RNA was isolated from the cells of each group. Afterward, the RNA sample was subjected to reverse transcription using the reverse transcription kit (Tiangen Biotechnology, Beijing, China). The 2 × SYBR Green qPCR Master Mix (Shanghai Dongsheng Biotechnology, Shanghai, China) was used. The internal control used in this study was *β-actin*. The relative expression of the gene was calculated using the 2-ΔΔCt technique. The primers utilized are given in Table 1.

**Table 1:** The primers utilized for overexpression of the gene

Gene name	Forward Primer (5′ → 3′)	Reverse Primer (5′ → 3′)
*IL17REL*	CAGGAGACGCAGTGTCAGAG	CAGGAGACGCAGTGTCAGA
*β-actin*	GGCTGTATTCCCCTCCATCG	CCAGTTGGTAACAATGCCATGT

### Cell culture and transfection

A375 (catalog number CL-0014) and A875 (catalog number CL-0255) were brought from Procell Life Science & Tech-nology.Co.,Ltd, and they were cultured in Dulbecco's Modified Eagle Medium (DMEM; Thermo Fisher Scientific, USA) supplemented with 10% fetal bovine serum (FBS). After adding the FBS, 100 U / mL of penicillin and 100 g / mL of streptomycin were introduced to ensure sterility. Both cell lines were incubated at standard growth conditions.

The plasmids overexpressing the *IL17REL* gene and their corresponding negative controls were obtained from Generay Biotech (China). Lipofectamine 2000 (Invitrogen, USA) was used for transfecting the human A375 and A875 cells.

### Measurement of the proliferation of cells

The BeyoClick™ EDU-55 cell proliferation detection kit (Beyotime, China) was prepared per the manufacturer's instructions. The kit provided a 5-Ethynyl-2′-deoxyuridine (Edu) solution from which a working solution was prepared and added to cells for 2 h. The cells were then fixed using a 4% paraformaldehyde solution and eventually treated with a 0.3% Triton X-100 permeability solution in a dark environment for 30 min. Hoechst nuclear fluorescence microscopy was used to detect EdU-stained cells.

### Measurement of intracellular ROS in cells

The production of intracellular reactive oxygen species (ROS) was quantified using a commercially available ROS detection kit (Beyotime, China). Concisely, 3 × 10^5^ cells were cultured in a 6-well plate and incubated overnight in standard growth conditions. The cells were subjected to staining using a concentration of 10 µM of DCFH-DA at 37 °C for 30 min. The images were taken and quantified.

### Colony formation assay

The A375 and A875 cell lines were seeded in a 6-well plate. Following a 14-day incubation period, the cells were treated with 100% methanol for fixation and subjected to staining with a 0.5% solution of crystal violet. Eventually, the colonies were systematically counted, and images were taken.

### Transwell assay

To assess the capacity of cells for transwell invasion, a volume of 100 µL containing 5 × 10^4^ cells in incomplete DMEM medium (serum-free) was introduced into transwell inserts (Corning, USA). As a nutritional attractant, 10% FBS was added to serum-free DMEM and put in the lower section of the transwell experiment. The cells on the bottom surface were preserved with 4% poly-formaldehyde (Beyotime, China) for 30 min after conducting a 16-h invasion experiment. Subsequently, for 30 min, these cells were stained with a 0.4% crystal violet solution (Beyotime, China). After removing cells from the top surface, the cells on the bottom were quantified by microscopic observation.

### Wound healing

The cells were gently scraped using a pipette, following the fusing of cellular components into a 6-well plate. Photographs were captured at the time points of 0 h and 24 h after the act of scratching.

### Statistical analysis

The R software (version 4.1.2) was used for statistical analysis. For the significant data analysis (like expression, infiltration ratio, and various eigenvalues, etc.), the two groups of samples were compared for differences via the Wilcoxon signed rank and compared differences between multiple groups of samples through the Kruskal–Wallis.

## Results

### Mutational characteristics associated with pTMB level in SKCM

**Fig. 1:**
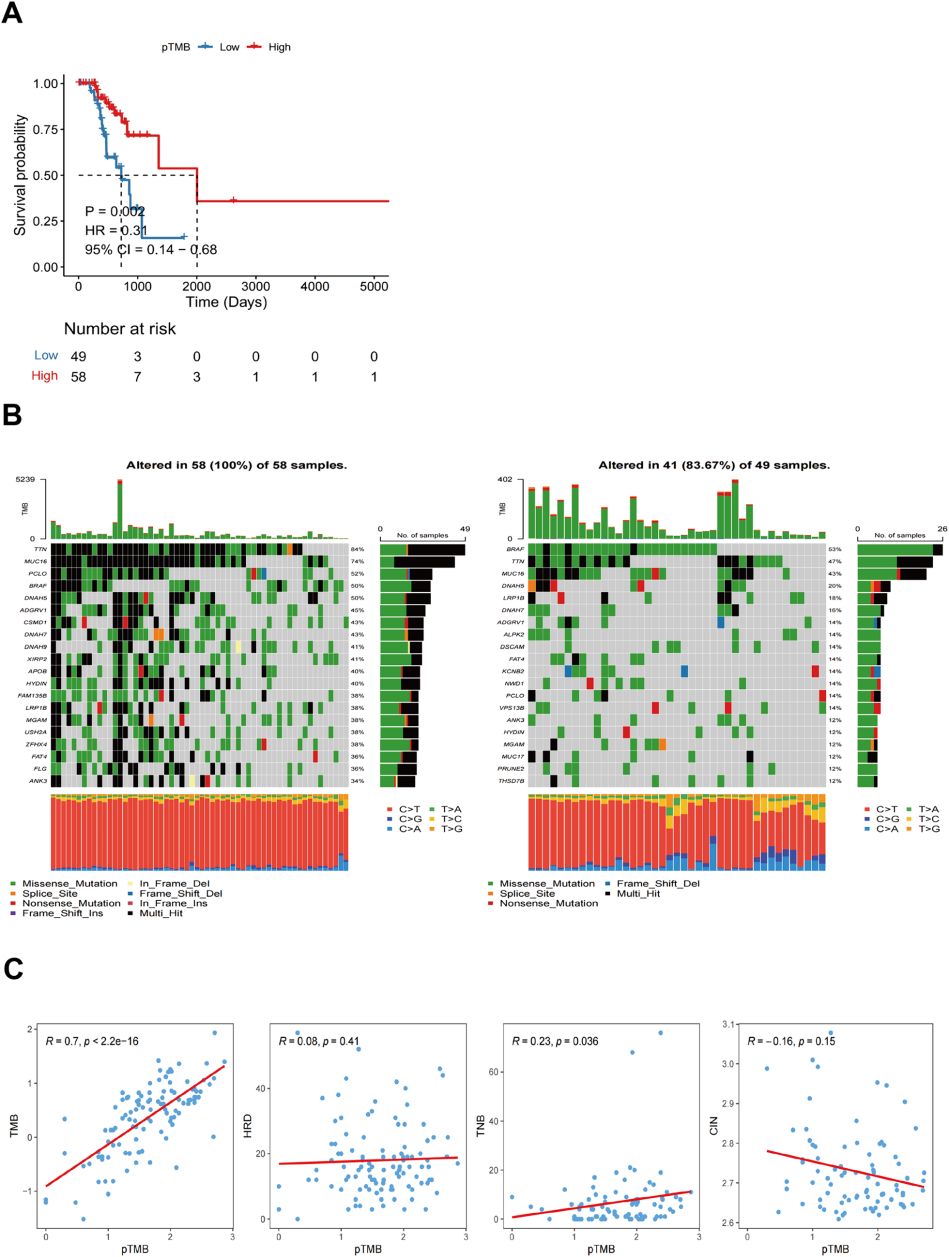
Shows the mutational characteristics linked to the pTMB level in SKCM. **A** Prognostic values of pTMB in SKCM. **B** Somatic mutations in different pTMB levels. (**C**) The relationship between different mutational markers and pTMB

Using an optimal cutoff value based on pTMB enables the differential classification of groups into L-pTMB and H-pTMB categories (Supplementary Table 1). The findings demonstrated that the H-pTMB subgroup showed a significantly greater survival rate than the L-pTMB subgroup, as shown in Fig. 1A. The proportion of gene mutations in the L-pTMB was much lower than in the H-pTMB, according to somatic mutation data of various pTMB levels (Fig. 1B). The correlation analysis of pTMB with TMB, HRD, TNB, and CIN was also performed, and its results indicated that TMB, HRD, and TNB were positively associated with pTMB. In contrast, pTMB was negatively associated with CIN (Fig. 1C). The distribution of clinicopathological features of pTMB and cutaneous melanoma showed significant differences between gender and pTMB (Supplementary Fig. 1A). This is unlike TMB, which was found to increase significantly with age regardless of gender in large sample data analyses (Li et al. 2022; Chalmers et al. 2017). This may indicate the advantage of pTMB in elderly CM.

### Immune characteristics associated with pTMB level in SKCM

**Fig. 2:**
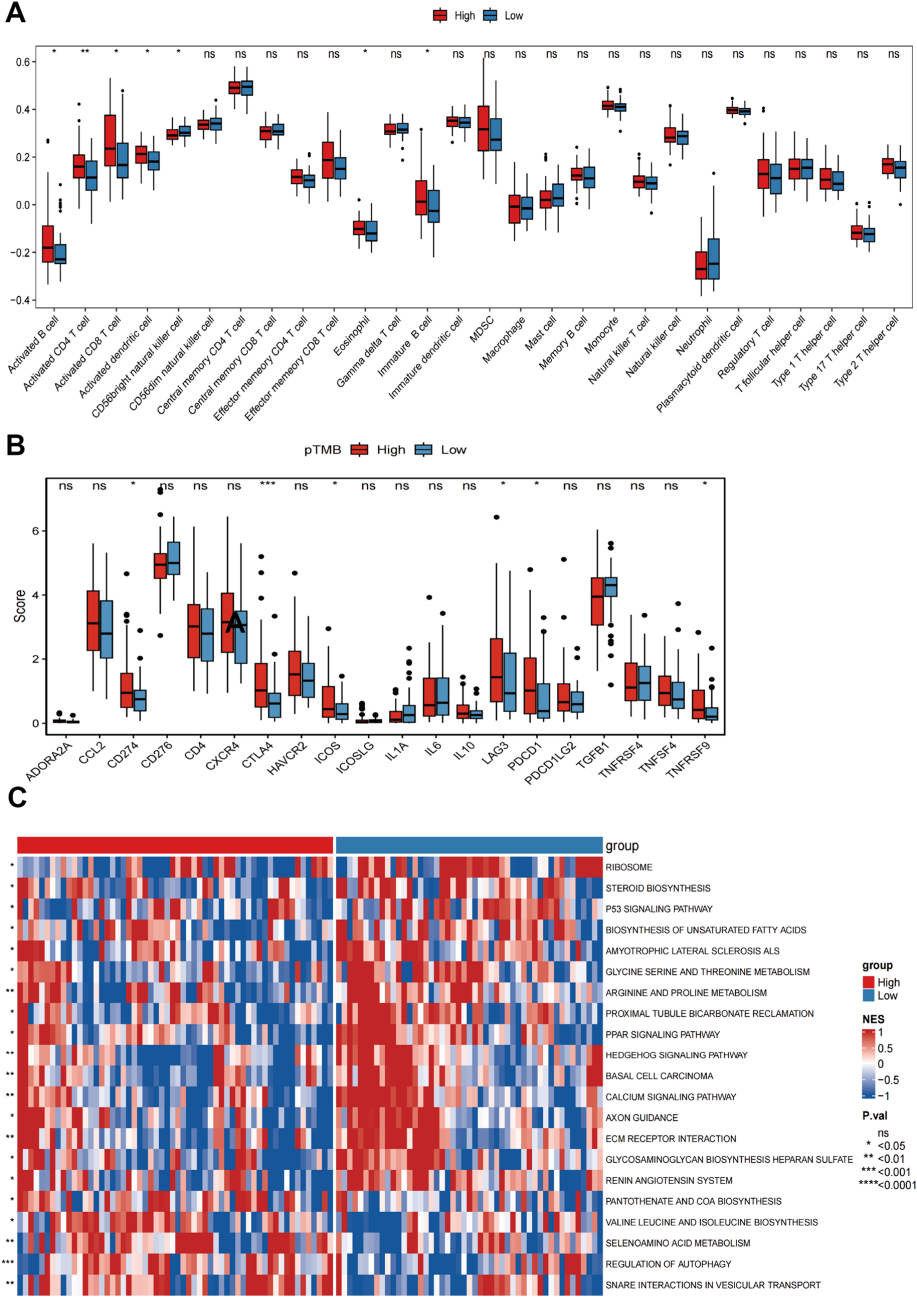
Immunological characteristics associated with pTMB concentration in SKCM. **A** ssGSEA immune infiltration with different pTMB levels. **B** Immune checkpoints with different pTMB levels. **C** GSVA of the immune pathway with varying levels of pTMB

The results of StromalScore, ImmuneScore, ESTIMATEScore, and TumorPurity for different pTMB levels were insignificant (Supplementary Fig. 1B). Immune cell subsets were quantified using the ssGSEA method. The findings revealed that the H-pTMB group had considerably more significant levels of infiltration of immature B cells, activated CD8^+^ T cells, activated CD4^+^ T cells, and other subsets (Fig. 2A). Upon evaluating the expression of *CD274*, *CTLA4*, and other immune checkpoint genes at different pTMB levels, it was seen that the H-pTMB subgroup showed a significantly higher expression of *CD274*, *CTLA4*, and *ICOS* (Fig. 2B). The H-pTMB group may thus be more responsive to immunotherapy, according to our inference. According to the GSVA differences, the H-pTMB group had a considerably high enrichment of the regulatory pathway of autophagy (Fig. 2C). Autophagy is directly linked to the control of the immune response in tumors. It is also essential for the proper function and survival of the immune system's effector and developmental T cells. The extracellular matrix (ECM)-receptor interaction pathway, linked to tumor resistance, was significantly higher in the L- pTMB group.

### Identification of pTMB-related signature

R software's "limma" package detected DEGs among the low-pTMB and high-pTMB subgroups. A comprehensive analysis revealed that a total of 2292 genes had differential expression patterns, with 1490 genes being up-regulated and 802 genes being down-regulated (Supplementary Table 2).

**Fig. 3:**
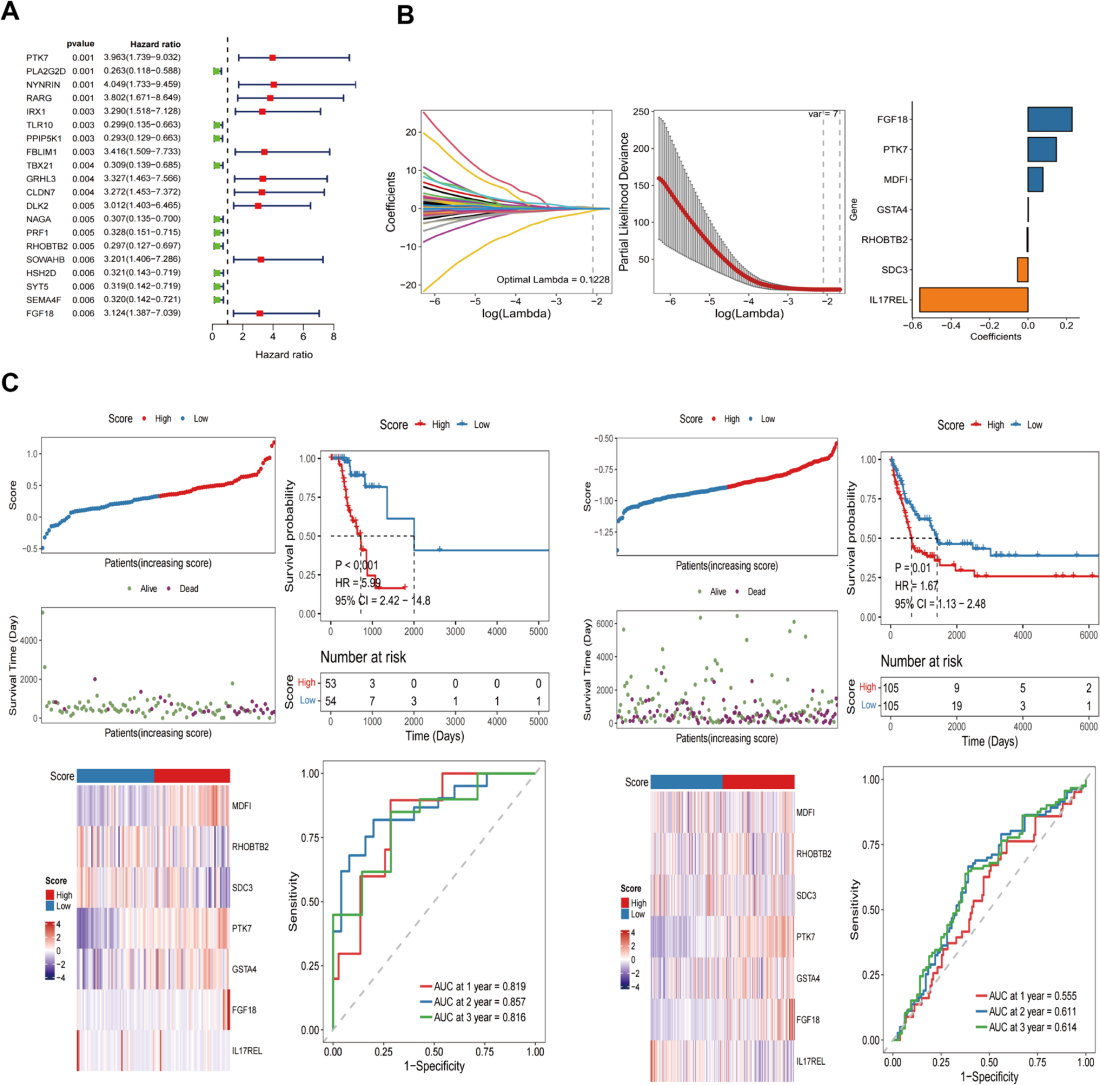
Establishing and validating the signature associated with pTMB. **A** Top 20 DEGs with prognostic values. **B** LASSO COX regression(with optimal lambda) identifying 7 host genes. **C** Patient statuses and expression patterns for seven host genes in the training cohort's high- and low-risk categories. The K-M survival curve and the AUC curve have distinct outcomes. **D** Status and expression patterns of seven host genes in the testing cohort's high- and low-risk patient groups. K-M survival curve and ROC curve demonstrating dissimilar outcomes

The univariate Cox regression analysis based on pTMB characteristics found 972 prognostic genes (*p* < 0.05). Following the intersection with differential genes, 217 genes having prognostic significance were found. The forest plot was constructed using the top 20 genes with the lowest *p*-values (Fig. 3A). Due to the extensive number of genes, which poses challenges for clinical identification, the LASSO regression model to refine the focus and determine the trajectory of the independent variables under study (Fig. 3B). As lambda increased, independent variable coefficients gradually decreased to zero. The RiskScore, which is the gene-based survival risk score model, was constructed using 7 LASSO-coefficient-carrying genes, including *IL17REL*, *SDC3*, *RHOBTB2*, *GSTA4*, *MDFI*, *PTK7*, and *FGF18*, based on the lambda value using LASSO. The confidence interval associated with each lambda value proves that the model achieves optimality when the number of genes is 7. This further supports the reliability of the candidate gene selection process. The RiskScore models were constructed using a ten-fold cross-validation approach, wherein the coefficients and expression levels of 7 specific genes were utilized to evaluate their influence on the OS outcome.

Based on median RiskScore, TCGA- SKCM cohort samples were divided into low- and high-risk categories (Supplementary Table 3). The KM survival analysis indicates that high-risk patients had a significantly lower OS than low-risk individuals. Additionally, the TCGA-SKCM cohort's OS could be predicted by the RiskScore, AUCs (area under the curve) for 1, 2, and 3 years were 0.819, 0.857, and 0.816, correspondingly (Fig. 3C).

Using the same technique as the validation set GSE65904, the RiskScore model created using the TCGA-SKCM cohort was assessed for stability. The results demonstrated that low-risk SKCM had more substantial survival benefits, consistent with the TCGA-SKCM cohort (Fig. 3C).

**Fig. 4:**
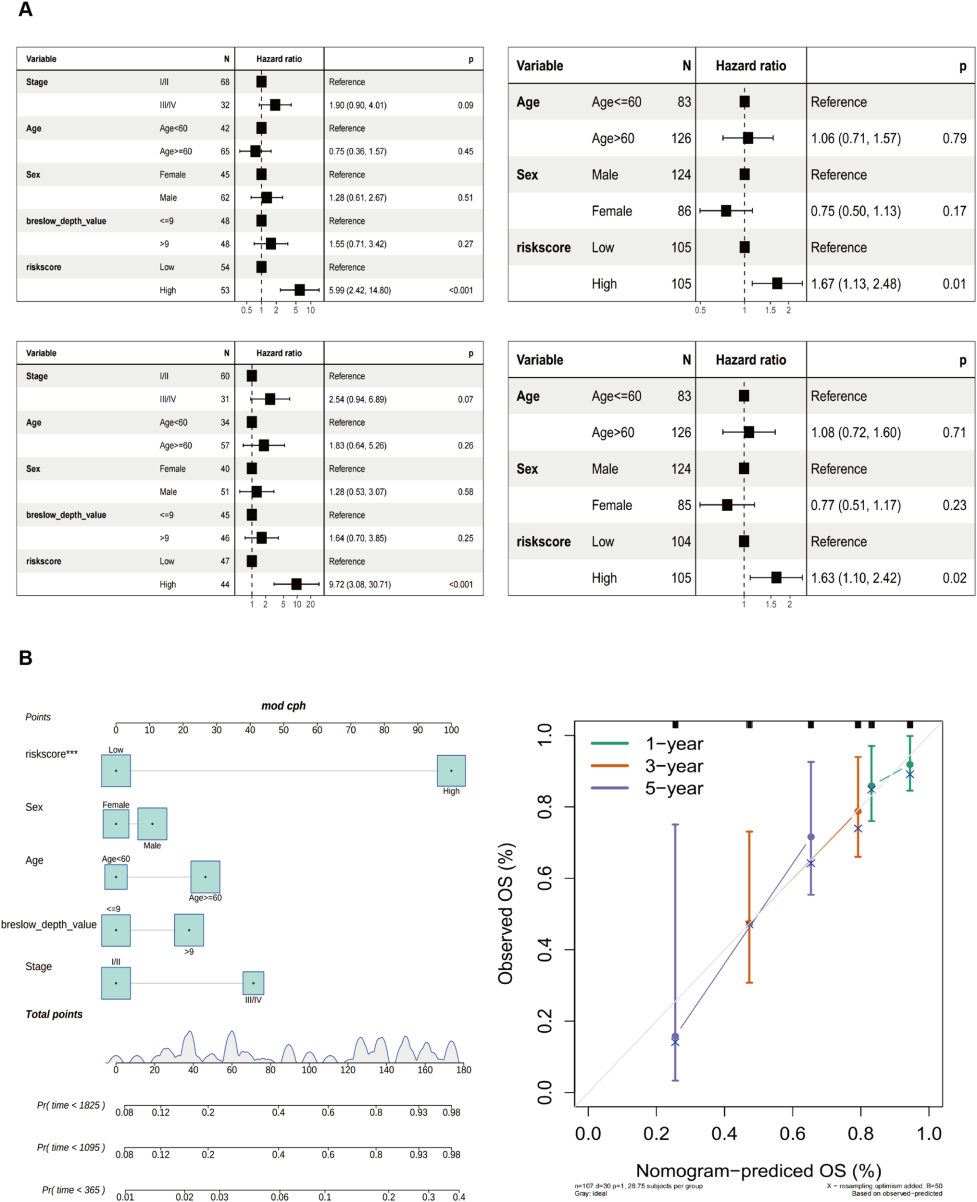
Development of the nomogram model. **A** Univariate and **B** multivariate Cox regression analyses for the risk score and other clinical variables of the training cohort. **C** Univariate and **D** multivariate Cox regression analysis for the risk score and other clinical characteristics of the testing cohort. **E** The construction of the nomogram aimed to develop a predictive model for estimating the probabilities of survival at 1-, 3-, and 5-year intervals. **F** Correction curve showing the consistency between predicted survival possibilities and observed survival rate

The univariate Cox regression analysis shows a strong association between TCGA-SKCM cohort risk score and OS (TCGA-SKCM cohort: HR = 5.99, 95% CI = 2.42–14.80, *p* < 0.001; GSE65904 cohort: HR = 1.67, 95% CI = 1.13–2.48, *p* = 0.01). Multivariate Cox regression analysis showed that risk score constituted an independent OS predictor (TCGA-SKCM cohort: HR = 9.72, 95% CI = 3.08–30.71, *p* < 0.001; GSE65904 cohort: HR = 1.63, 95% CI = 1.10–2.42, *p* = 0.02) (Fig. 4A). A detailed investigation of the clinical applicability of the risk score signature. The present study observed that no clinical indicators exhibited positive findings in the independent prognostic analysis. Consequently, all the clinical indicators and risk scores were utilized to construct nomogram models and generate a calibration curve. The results are depicted in Fig. 4B.

### Differences in biological characteristics between the prognostic signature low- and high-risk groups

The biological traits of low- and high-risk subgroups were assessed using GSVA.

The enrichment proportion of basal cell carcinoma patients in the high-risk group was significantly higher than in the low-risk group.

**Fig. 5:**
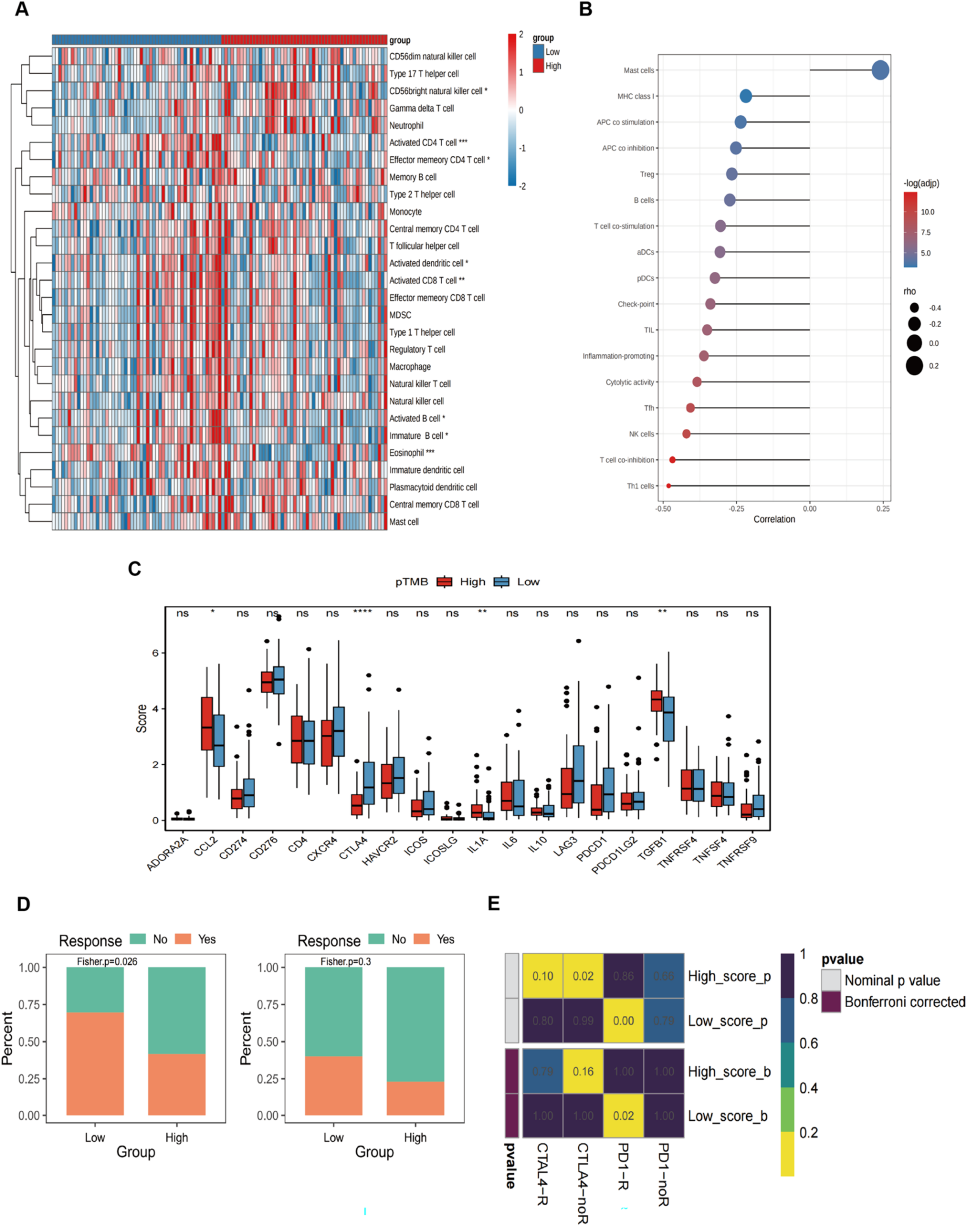
Differences in the biological features of the pTMB-related signature

The enrichment fraction was significantly higher in the high-risk subgroup than in the low-risk subgroup in basal cell carcinoma, melanogenesis, and other pathways (Fig. 5).

### Immune-associated characteristic differences and prediction of potential drug therapy between the prognostic signature low- and high-risk groups

**Fig. 6:**
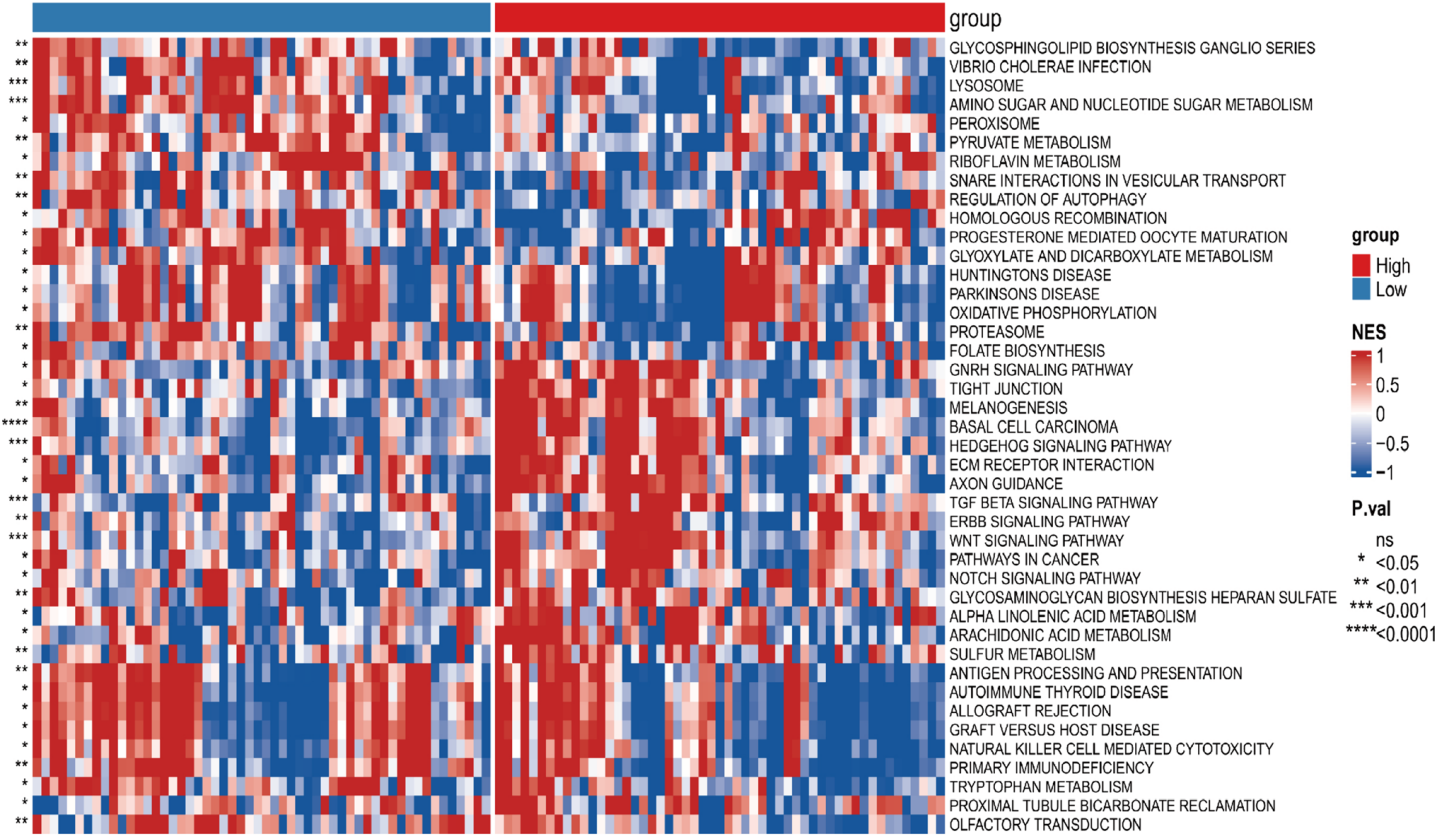
Variations in immune-related characteristics and possible drug treatment prediction in high-risk and low-risk populations. **A** Immune cell infiltration in high-risk and low-risk groups. **B** The graph displays the variations in immune function features between the low-risk and high-risk cohorts. **C** Boxplot showing immune checkpoint expression differences between low- and high-risk SKCM patients. **D** Drug sensitivity in low- and high-risk SKCM, including CTLA4 and PD-1. **E** Heat map for the response possibility of anti-PD-1 and anti-CTLA-4 treatment in the two risk groups

Several immune cell subsets were quantified using ssGSEA, showing that high-risk subgroups had more CD56^bright^ natural killer cell infiltration. In contrast, the activated CD4^+^ T cells and eosinophils invaded the low-risk subgroup (Fig. 6A). The difference in the immune function characteristics revealed that the levels of mast cells were considerably elevated in the high-risk subgroup. On the other hand, the low-risk subgroup had more NK cells, T cell co-inhibition, and Th1 cells (Fig. 6B). Checkpoint expression analysis showed a statistically significant increase of *CTLA4* in the low-risk subgroup (Fig. 6C).

The melanoma treatment-associated sensitivity analysis conducted on the low- and high-risk subgroups indicates that the low-risk subgroup exhibited more positive responses to CTLA4 and PD-1 therapy than the high-risk subgroup (Fig. 6D). Figure 6E shows that the Submap approach compared the immunotherapy efficacy. The findings indicated a substantial similarity between the low-risk subgroup and the data from PD-1 treatment, implying that this group exhibited sensitivity to immunotherapy.

### Expression of signature genes in SKCM

**Fig. 7:**
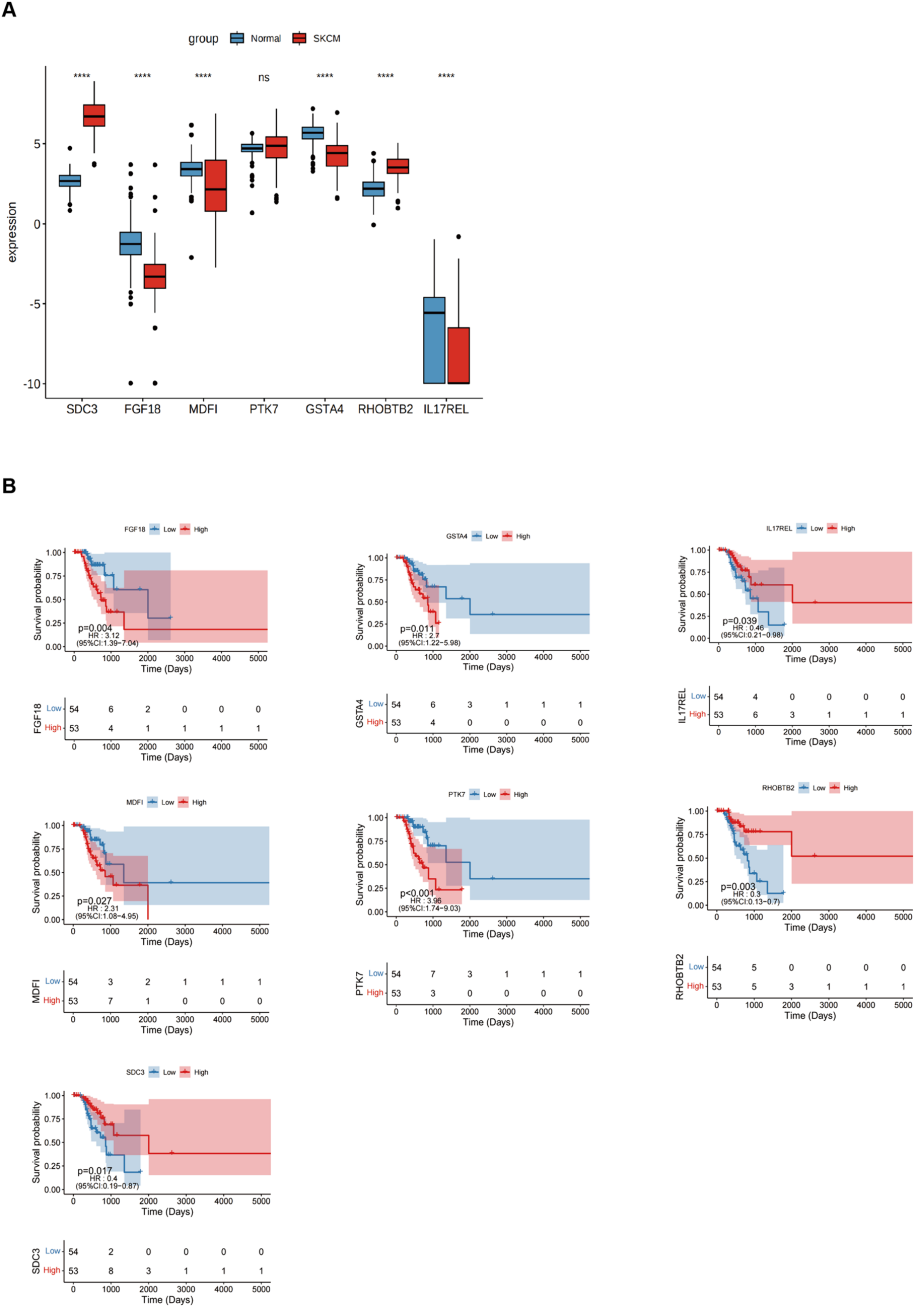
Expression of signature genes in SKCM. **A** Differentially expressed genes were evaluated on samples obtained from both normal and malignant tissues. **B** The K-M survival curve was produced to analyze the 7 host genes

The differentially expressed genes were evaluated in normal and tumor samples to validate the risk-scoring model. For this, seven potential genes were investigated. The findings showed that in SKCM, the expression of *FGF18*, *MDFI*, *GSTA4*, and *IL17REL* were lowered. In contrast, SKCM increased the expression levels of the remaining two genes, except for *PTK7*, which did not show a statistically significant difference (Fig. 7A). The prognostic value of 6 genes in SKCM was analyzed and revealed that just the *IL17REL* gene exhibited expression levels in SKCM that correlated with OS. Specifically, it was shown that *IL17REL* expression was relatively low in SKCM cases, indicating a poor prognosis (Fig. 7B). Therefore, validation of a prognostic signature by examining the effect of the *IL17REL* gene in tumor tissues and cell lines was done.

### Biological functions of the selected gene

**Fig. 8:**
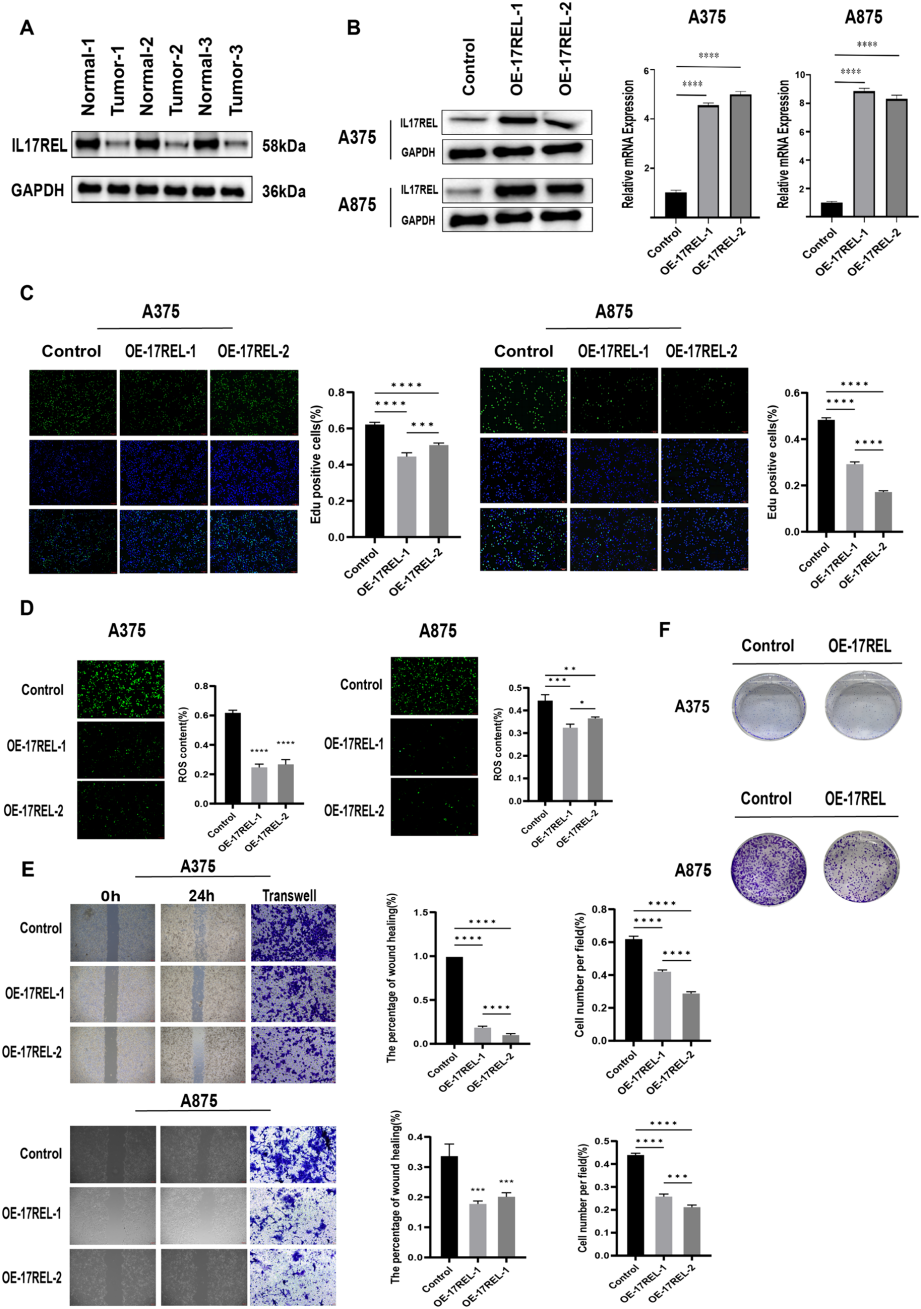
*IL17REL* inhibited the proliferation, clone, and migration in vivo and in vitro. **A** The expression of *IL17REL* was down-regulated in CM samples. **B** *IL17REL* was overexpressed in A375 and A875 cells. **C** OE-IL17REL inhibited proliferation in A375 and A875 cells.(**D** OE-IL17REL decreases ROS in A375 and A875 cells. **E**, **F** OE-IL17REL inhibited migration, invasion, and clone in A375 and A875 cells

*IL17REL* expression was detected in 3 in situ collected CM tissues, and the results from western blot (WB) showed a decrease in *IL17REL* gene expression, consistent with the bioinformatics analysis (Fig. 8A). To further verify the effect of the *IL17REL* gene on the prognosis of CM, the overexpression efficiency of the *IL17REL* gene in human A375 and A875 cells was conducted using qRT-PCR and WB (Figure), and OE—IL17REL-1 and OE—IL17REL-2 were selected for further investigations (Fig. 8B). The findings from the EdU experiment demonstrated that the upregulation of *IL17REL* had a detrimental effect on the proliferative capacity of malignant melanoma (MM) cells (Fig. 8C). The levels of ROS generation in MM cells exhibited a reduction after the overexpression of *IL17REL* (Fig. 8D). In addition, it was observed that the overexpression of *IL17REL* resulted in the suppression of MM cell migration, as evidenced by the findings from wound healing and transwell experiment (Fig. 8E). The A375 and A875 cells overexpressed *IL17REL* had significantly higher cell proliferation and colony formation than the normal control cells (Fig. 8F).

## Discussion

Melanoma exhibits a notable degree of immunogenicity owing to its elevated load of genetic mutations and neoantigens, which can potentially trigger the commencement of the tumor cell elimination phase (Kalaora et al. 2022; Puig-Saus et al. 2023). The correlation between tumor mutation burden and the response to ICB has been widely acknowledged, making it a valuable predictive technique for assessing the outcomes of patients undergoing ICB treatment (Liu et al. 2019). The immunoediting theory suggests that, when subjected to immunotherapy, cancer cells acquire resistance against effector immune cells by favoring the growth of clones with reduced immunogenicity. According to a study, neoantigen loss was seen as a result of either the removal of tumor subclones or the loss of copy numbers (Łuksza et al. 2022). The observed loss was associated with the emergence of acquired resistance to immune checkpoint therapy (Anagnostou et al. 2017). In summary, these findings demonstrate the importance of accurately forecasting the clonality and heterogeneity of neoantigens.

Niknafs et al., 2023, reported a collection of mutations characterized as "persistent" because of their reduced susceptibility to lose or develop immunoediting during tumor progression. These mutations primarily manifest as genomic and chromosomal deletions, constituting a minor proportion (10%) of somatic mutations. They defined it as pTMB, which refers to the cumulative count of single-copy and multi-copy mutations. The Whole Exome Sequencing (WES) research conducted on a collection of tumor samples both before and after ICB treatment revealed that persistent mutations exhibited a reduced tendency to induce subclonal loss during tumor progression within ICB (Davoli et al. 2017). Furthermore, there was no association observed between tumor clonal heterogeneity and the presence of persistent mutations. The researchers assessed the varying reclassification of cancer in 33 distinct tumor types. The results revealed that the average reclassification rate for the low/ high TMB subgroup, compared to the persistent low/high TMB subgroup, was 33%. This suggests that TMB and pTMB varied across all forms of cancer. In clinical applications, pTMB is better than TMB in predicting ICB response, and the authors further suggest that the predictive capacity of TMB to clinical outcomes primarily depends upon persistent mutations. The measurement of pTMB is an innovative method and has potential application in predicting ICB treatment outcomes in CM.

The present investigation examined the clinical and immunological associations between pTMB and CM, identified prognostically significant differentially expressed genes based on pTMB characteristics, developed a risk scoring system using pTMB-related gene modules, and investigated their prognostic utility, biological distinctions among various groups, and immune attributes for predicting potential therapeutic strategies for CM. Combined with candidate genes' differential expression and predictive value, further validation of the model in tissue specimens and in vitro experiments was done.

Among the pTMB-associated immune features, immature B cells, activated B cells, activated CD8^+^ T cells, and activated CD4^+^ T cells in the high- pTMB subgroup were up-regulated. Studies have shown that activated CD8^+^ and CD4^+^ T cells boost anti-tumor immunity. These cells are crucial in promoting the density and targeting of CD4^+^/CD8^+^ effector T cells, enhancing immunotherapy's efficacy (Hirschhorn et al. 2023; Virassamy et al. 2023). There is a correlation between elevated levels of B-cell infiltration within the tumor microenvironment and favorable clinical outcomes in melanoma patients who undergo immunotherapy (Cabrita et al. 2020; Helmink et al. 2020). A comparison of immune checkpoint gene expression across multiple pTMB modes was performed. *CD274*, *CTLA4*, and *ICOS* exhibited a statistically significant upregulation in the high-pTMB subgroup.

Moreover, the high-pTMB subgroup showed a substantial rise in the regulatory pathway of autophagy, as seen by the GSVA results of various pTMB modes. Autophagy is essential for immune system development and affects T cell survival and function, influencing the control of immunological responses against tumors (Xia et al. 2021; Debnath et al. 2023). The ECM-receptor interaction pathway exhibited a significant enrichment in the group with low—p TMB. This enrichment was found to be connected with the development of drug resistance in tumors (Holle et al. 2016). Based on the study mentioned above, it is postulated that individuals with CM who exhibit high levels of pTMB may potentially be more sensitive to immunotherapy.

The current research used a risk score based on pTMB gene expression in multiple modes to construct a prognosis model and identified the differential genes with prognostic significance using analysis of different features. The results demonstrated this model's efficacy in accurately predicting patients' OS. Also, a decreased expression of the *IL17REL* gene was linked to worse outcomes in individuals with CM. This finding supports the validation of our proposed prognostic signature from a clinical standpoint. *IL17REL* encodes an IL17RE-like protein, and IL17RE is the least understood member of the IL17R family. In genome-wide association (GWAS) studies (Franke et al. 2010) and whole exon sequencing studies (Hu et al. 2021), *IL17REL* was found to be strongly correlated with the development of inflammatory bowel disease (IBD). Recent evidence indicates a strong association between *IL17REL* and the prognostic evaluation of HPV- associated head and neck squamous cell cancer (Yanan et al. 2023; Sun, et al. 2023). There needs to be more understanding regarding the expression and role of *IL17REL* in many cell types, particularly in tumor cells. Our study contributes novel insights to the realm of melanoma research.

In summary, this study has effectively developed and validated a predictive model linked to pTMB and has confirmed the possible impact of *IL17REL* on OS in individuals with melanoma. However, more experimental investigations are required to elucidate the precise molecular mechanism behind this association. Due to insufficient patient data, the study was incomplete regarding pre- and post-immunotherapy data for both the low and high-risk subgroups. Thus, the predictive models of pTMB can enhance the accuracy of patient survival predictions and serve as a valuable foundation for personalized decision-making in clinical settings.

### Supplementary Information

Below is the link to the electronic supplementary material.

Supplementary file1 Supplementary Figure 1 A The distribution of clinicopathological features of pTMB levels and cutaneous melanoma, B The results of StromalScore,ImmuneScore, ESTIMATEScore, and TumorPurity for different pTMB levels (PNG 172 KB)

Supplementary file2 Supplementary Table 1: The differential classification of groups into L-pTMB and H-pTMB categories. (XLSX 14 KB)

Supplementary file3 Supplementary Table 2: The DEGs among the low-pTMB and high-pTMB subgroups. (TXT 2447 KB)

Supplementary file4 Supplementary Table 3: The differential classification of groups into low- and high-risk categories. (XLSX 1857 KB)

## Data Availability

The data used to support the findings of this study are available from the corresponding author upon request.
