# Supplementary figures and images for: Developing a prognostic model for skin melanoma based on the persistent tumor mutation burden and determining IL17REL as a therapeutic target
Source: J Cancer Res Clin Oncol. 2024 Jun 20;150(6):313. doi: 10.1007/s00432-024-05843-x (PMC11189994; doi:10.1007/s00432-024-05843-x)

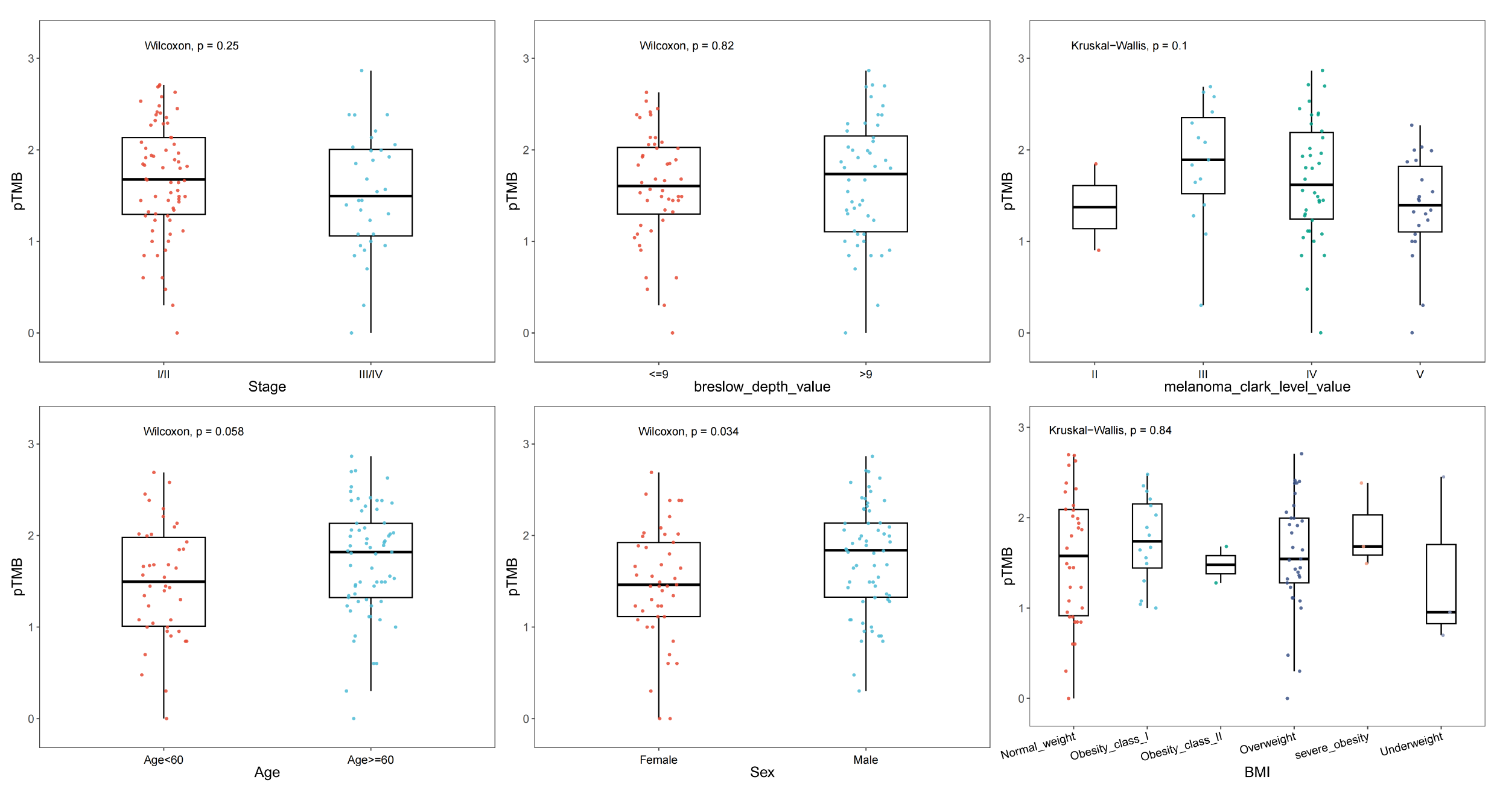

Supplement: Supplementary file 1 — Supplementary file1 Supplementary Figure 1 A The distribution of clinicopathological features of pTMB levels and cutaneous melanoma, B The results of StromalScore,ImmuneScore, ESTIMATEScore, and TumorPurity for different pTMB levels (PNG 172 KB) [file 432_2024_5843_MOESM1_ESM.png]
